# Efficient Schottky Junction Construction in Metal‐Organic Frameworks for Boosting H_2_ Production Activity

**DOI:** 10.1002/advs.202004456

**Published:** 2021-05-07

**Authors:** Yang Wang, Wei Zhang, Dan Li, Jianping Guo, Yu Yu, Kejian Ding, Wubiao Duan, Xiyou Li, Heyuan Liu, Pengkun Su, Bo Liu, Jianfeng Li

**Affiliations:** ^1^ College of Materials Science and Opto‐electronic Technology, CAS Center for Excellence in Topological Quantum Computation & Center of Materials Science and Optoelectronics Engineering University of Chinese Academy of Sciences Yanqi Lake, Huairou District Beijing 101408 P. R. China; ^2^ Department of Chemistry, School of Science Beijing Jiaotong University Beijing 100044 P. R. China; ^3^ State Key Laboratory of Solid Waste Reuse for Building Materials Beijing Building Materials Academy of Science Research Beijing 100041 P. R. China; ^4^ School of Material Science and Engineering China University of Petroleum (East China) Qingdao Shandong 266580 China

**Keywords:** encapsulation, facets selection, metal‐organic frameworks, PtPd alloys, Schottky junctions, UiO‐66‐NH_2_

## Abstract

Manipulation of the co‐catalyst plays a vital role in charge separation and reactant activation to enhance the activity of metal‐organic framework‐based photocatalysts. However, clarifying and controlling co‐catalyst related charge transfer process and parameters are still challenging. Herein, three parameters are proposed, *V*_transfer_ (the electron transfer rate from MOF to co‐catalyst), *D*_transfer_ (the electron transfer distance from MOF to co‐catalyst), and *V*_consume_ (the electron consume rate from co‐catalyst to the reactant), related to Pt on UiO‐66‐NH_2_ in a photocatalytic process. These parameters can be controlled by rational manipulation of the co‐catalyst via three steps: i) Compositional design by partial substitution of Pt with Pd to form PtPd alloy, ii) location control by encapsulating the PtPd alloy into UiO‐66‐NH_2_ crystals, and iii) facet selection by exposing the encapsulated PtPd alloy (100) facets. As revealed by ultrafast transient absorption spectroscopy and first‐principles simulations, the new Schottky junction (PtPd (100)@UiO‐66‐NH_2_) with higher *V*_transfer_ and *V*_consume_ exhibits enhanced electron‐hole separation and H_2_O activation than the traditional Pt/UiO‐66‐NH_2_ junction, thereby leading to a significant enhancement in the photoactivity.

## Graphical abstract

An efficient Schottky junction PtPd cubes@UiO‐66‐NH_2_ is constructed by optimizing the *V*_transfer_, *D*_transfer_, and *V*_consume_, respectively. When compared to pristine UiO‐66‐NH_2_ and the traditional Schottky junction (Pt/UiO‐66‐NH_2_), PtPd cubes@UiO‐66‐NH_2_ with high *V*_consume_ and *V*_transfer_ show a >116‐ and 4.4‐fold enhancement in H_2_ generation, respectively.

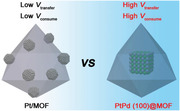

## 1 Introduction

Metal‐organic frameworks (MOFs) have drawn significant attention over the past two decades as efficient photocatalysts for H_2_ production due to their unique structural diversity, ordered porous structures, and channel tailoring characteristics.^[^ ^1^, ^2^, ^3^, ^4^ ^]^ Their semiconductor behavior was confirmed in 2007, which showed that the organic linkers in MOF‐5 can be photoexcited to generate charge and allow electron transfer to metal‐oxo clusters to complete the separation of the electrons and holes (i.e., ligand to cluster charge transfer mechanism).^[^ ^5^ ^]^ Upon comparison to semiconductor‐based photocatalysts, the organic linkers can be regarded as the valance band (VB) and the metal‐oxo cluster as the conduction band (CB).^[^ ^6^ ^]^ Therefore, the photoactivities of MOFs are mainly decided by their i) light harvesting ability, ii) charge transfer and separation efficiency, and iii) reactant (e.g., H_2_O) adsorption and activation abilities. Notably, for the construction of light harvesting MOFs, strategies such as grafting light adsorbing functional groups (e.g., ‐NH_2_) to the organic linker,^[^ ^7^, ^8^ ^]^ conjugating dyes (e.g., Rhodamine B and Erythrosin B) to MOFs,^[^ ^9^, ^10^, ^11^ ^]^ and the introduction of plasmonic metals (e.g., Au)^[^ ^12^, ^13^ ^]^ have been recently developed. However, the remaining two issues of (ii) and (iii), to the best of our knowledge, are yet to be addressed due to the high charge recombination rate and difficulty in controlling the interactions between the reactant and MOFs.

It is known that constructing a Schottky junction on photocatalysts is an effective way to inhibit electron‐hole recombination, particularly when using a noble metal such as Pt because Pt possesses a high work function and low overpotential, which can form a Schottky barrier with the photocatalyst.^[^ ^14^, ^15^ ^]^ The Schottky barrier facilitates electron trapping on Pt and can prevent electrons from flowing back to the photocatalyst to some extent, resulting in efficient electron‐hole separation. Despite substantial progress in the use of Pt to construct a conventional Schottky junction (e.g., the most commonly used Pt/photocatalyst system), several challenges remain; these include high‐cost and substantial Pt loss during cyclic testing. Most importantly, the following parameters of the charge recombination process related to Pt are not well understood and cannot be controllably designed: *V*_transfer_ (the electron transfer rate from MOF to co‐catalyst), the electron transfer rate from MOFs to Pt, which has been generally regarded to significantly influence the electron‐hole recombination efficiency;^[^ ^13^, ^16^, ^17^, ^18^, ^19^, ^20^, ^21^, ^22^, ^23^, ^24^ ^]^ *D*_transfer_ (the electron transfer distance from MOF to co‐catalyst), the distance for electron transfer from MOFs to Pt, which determines the final amount of available charge after the long‐distance transfer process;^[^ ^16^ ^]^ *V*_consume_ (the electron consume rate from co‐catalyst to the reactant), the rate of electron transfer from Pt to the reactant for activation, which reflects the efficiency of electron use and the photoreduction efficiency.

**Scheme 1:**
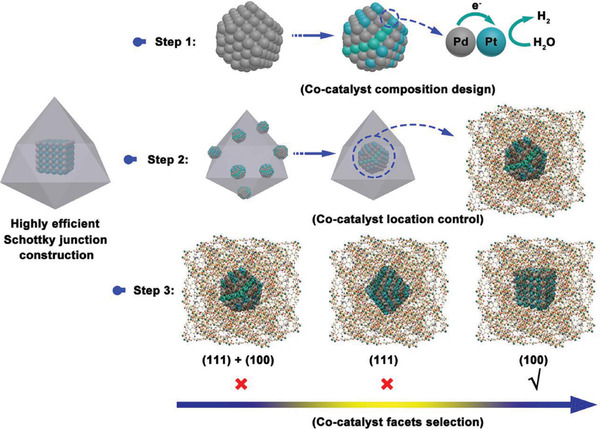
Schematic illustration of the construction of a highly efficient MOF‐based Schottky junction.

Herein, we report the construction of a consecutive Schottky junction via rational control over these three important parameters, *V*_transfer_, *D*_transfer_, and *V*_consume_. This engineering approach aims to simultaneously inhibit electron‐hole recombination and activate the reactant, which can be achieved by manipulating the co‐catalyst of Pt in MOFs using three steps (**Scheme** **1**): i) Partial substitution of Pt with Pd to form PtPd alloy, which adds a one electron transfer pathway to increase the *V*_transfer_ and reduce the cost of the co‐catalyst simultaneously, ii) encapsulation of the PtPd alloy into the MOFs to reduce the electron transfer distance (*D*_transfer_) to substitute the traditional electron transfer pathway from the interior to the external surface of the photocatalyst and further increase the *V*_transfer_, and iii) manipulation of the encapsulated PtPd alloy facets to enhance the reactant activation step (i.e., H_2_O molecules) and increase the *V*_consume_ for the faster consumption of electrons.

## 2 Results and Discussion

**Figure 1:**
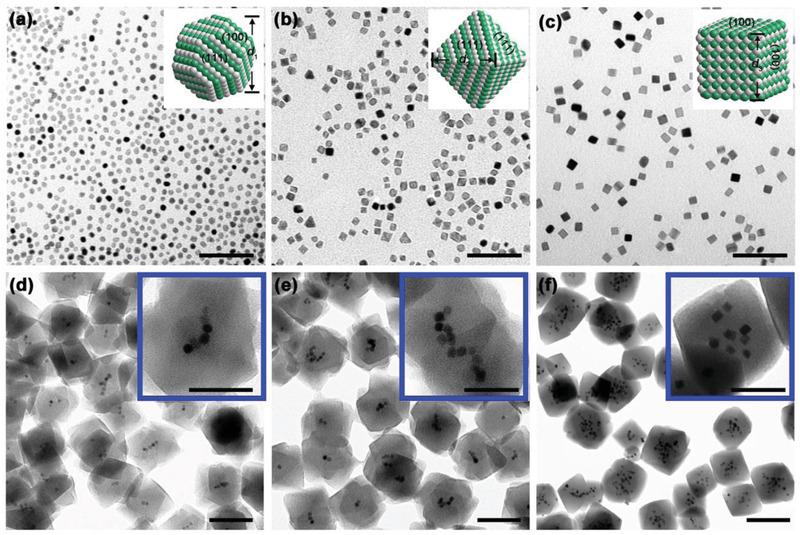
a)TEM images of PtPd polyhedrons, b) PtPd octahedrons, c) PtPd cubes. d) PtPd polyhedrons@UiO‐66‐NH_2_, e) PtPd octahedrons@UiO‐66‐NH_2_, and f) PtPd cubes@UiO‐66‐NH_2_. Scale bars for (a–c): 50 nm and (d–f): 100 nm. The insets of (a–c) are the corresponding model structures. *d* is the particle size used for calculating the external surface area and the volume of a single particle. Scale bars for (d–f): 50 nm.

Amino grafted UiO‐66 (i.e., UiO‐66‐NH_2_) was selected as the proof of concept MOF‐based photocatalyst due to its enhanced light harvesting ability and stability toward water when compared to UiO‐66. In the first step, the proposed co‐catalyst of polyhedral PtPd alloy (**Figure** **1**a and Figure S1a, Supporting Information) with an average diameter of *d*_1_ = 5.4 nm and elemental molar ratio of Pt: Pd = 1:1, which was determined using inductively coupled plasma mass spectrometry (ICP‐MS) (**Table** **1**) was prepared via the partial substitution of Pt by Pd. The alloy structure was confirmed by X‐ray diffraction (XRD) analysis. All the as‐prepared PtPd samples exhibit a typical *fcc* structure, identical to pure Pt (JCPDF 04‐0802) and Pd (JCPDF 46‐1043) particles and the main peaks observed in range of 2*θ* = 40°–90° correspond to (111), (200), (220), and (311) respectively, as shown in Figure S2a, Supporting Information. As can be seen in the magnified view of the (111) plane (Figure S2b, Supporting Information), all the (111) peaks of the PtPd particles are symmetrical and lie between Pt and Pd, which indicates the formation of the PtPd alloy.^[^ ^25^ ^]^ To compare their catalytic performance, the same molar ratio of pure Pt polyhedrons (particle size = 5.4 nm, Figure S1b, Supporting Information) and PtPd alloy (i.e., n(Pt + Pd)_PtPd polyhedrons_ = n(Pt)_Pt_) were mixed with the same amount of UiO‐66‐NH_2_ to construct PtPd polyhedrons/UiO‐66‐NH_2_ and Pt/UiO‐66‐NH_2_ Schottky junctions, respectively, which are shown in Figure S3, Supporting Information (≈0.5 wt% Pt/UiO‐66‐NH_2_ loading). For a standard H_2_ production test, 10 mg of the photocatalyst was dispersed in 101 mL of a solution of CH_3_CN, H_2_O, and triethanolamine (TEOA) (90:1:10 v/v/v) and the resulting suspension was then transferred into the photocatalyst evaluation system (Figure S4, Supporting Information) to produce H_2_, which was monitored periodically using gas chromatography.

**Table 1:** Summary of the co‐catalyst parameters and H_2_ evolution performance of Pt and PtPd alloy hybridized with UiO‐66‐NH_2_

	*D* ^a)^ [nm]	*S* ^b)^ [nm^2^]	*V* ^c)^ [10^−21^ cm^3^]	*ρ* ^d)^ [g cm^−3^]	*M*_1_ ^e)^ [10^−21^g]	*M*_2_ ^f)^ [10^−6^g]	*N* ^g)^ [10^12^]	H_2_ evolution [µmol h^−1^]	TOF_specific_ ^h)^ [min^−1^ nm^−2^]
Pt/UiO‐66‐NH_2_	5.4	91.6	82.4	21.45	1767.5	47.8	27.0	1.59	6.4
PtPd polyhedrons/UiO‐66‐NH_2_	5.4	91.6	82.4	16.80	1384.3	23.2 + 12.8	26.0	2.55	10.7
PtPd polyhedrons @UiO‐66‐NH_2_	5.4	91.6	82.4	16.80	1384.3	23.6 + 13.2	26.6	5.36	22.1
PtPd octahedrons @UiO‐66‐NH_2_	6.6	150.9	135.5	16.80	2276.4	24.2 + 13.2	16.4	4.53	18.4
PtPd cubes @UiO‐66‐NH_2_	7.8	365.0	474.6	16.80	7973.3	23.6 + 12.6	4.5	6.97	42.6

^a)^ Average diameter of co‐catalysts

^b)^ External surface area of a single co‐catalyst particle

^c)^ Volume of a single particle

^d)^ Density of a single particle

the density (*ρ*) of PtPd alloy was calculated by the formula of (*m*_1_ + *m*_2_)/(*m*_1_/*ρ*_1_ + *m*_2_/*ρ*_2_)

^e)^ Mass of a single particle

^f)^ The amount of co‐catalyst used in each H_2_ evolution test, which was detected using ICP‐MS

the mass of Pt and Pd in PtPd polyhedrons/UiO‐66‐NH_2_ was 23.2 × 10^−6^ g and 12.8 × 10^−6^ g, respectively

^g)^ The total number of particles participating in the reaction

^h)^ The calculated number of H_2_ molecules generated per nm^2^ per minute based on the surface of a single co‐catalyst particle.

**Figure 2:**
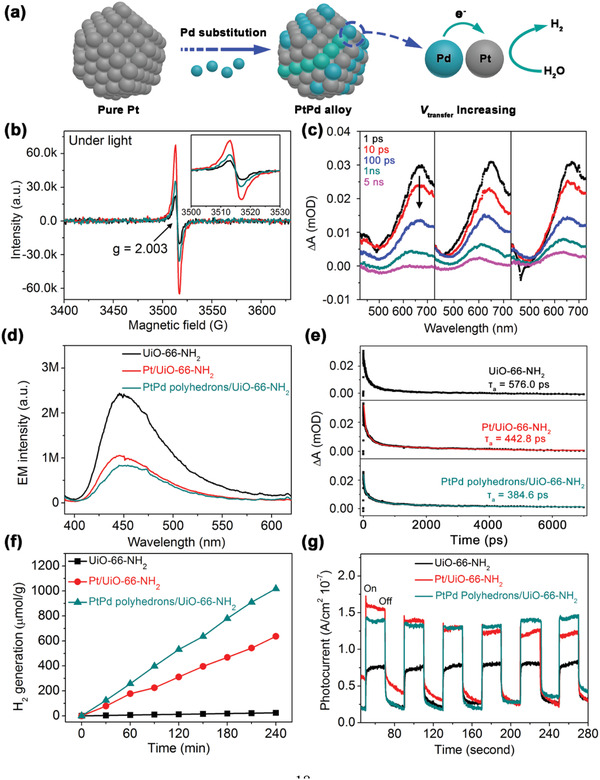
a) Schematic illustration of step 1, b) ESR signal comparison under visible light irradiation for 5 min, c) TA spectra pump at 380 nm at different delays in the range of 440−730 nm, d) steady‐state PL emission spectra excited at 380 nm, e) TA kinetics observed at a wavelength of 650 nm and corresponding multi‐exponential fitting, f) H_2_ production performance, and g) photocurrent measurements for pristine UiO‐66‐NH_2_, Pt/UiO‐66‐NH_2,_ and PtPd polyhedrons/UiO‐66‐NH_2_.

It should be noted that the proposed *V*_transfer_ parameter (**Figure** **2**a) is actually composed of two parts: *V*_transfer‐1_ (defined as the electron transfer rate from the linker to the Zr‐O cluster) and *V*_transfer‐2_ (defined as the electron transfer rate from the Zr‐O cluster to co‐catalyst), as shown in Figure S5, Supporting Information, which will be separately investigated. Considering a semiconductor for comparison, *V*_transfer‐1_ can be regarded as the electron transfer process for photoexcited electrons from the VB to CB, which highly depends on the light adsorption ability and can therefore be determined using UV–vis spectroscopy. Figure S6, Supporting Information shows that the *E*_g_ values have the following order: Pt/UiO‐66‐NH_2_ < PtPd polyhedrons/UiO‐66‐NH_2_ < UiO‐66‐NH_2_, which suggests that Pt/UiO‐66‐NH_2_ exhibits the best photoexcitation process, or in other words, the highest *V*_transfer‐1_ as can be seen in the in situ electron spin resonance (ESR) spectra (Figure 2b and Figure S7, Supporting Information). Under the same conditions, Pt/UiO‐66‐NH_2_ exhibits the highest signal intensity (intensity order: Pt/UiO‐66‐NH_2_ > PtPd polyhedrons/UiO‐66‐NH_2_ > UiO‐66‐NH_2_), which indicates that the generation rate of Zr^3+^ in the original Zr^4+^‐O clusters^[^ ^26^, ^27^, ^28^ ^]^ was the fastest. Since both *V*_transfer‐1_ and *V*_transfer‐2_ can lead to a valence change in the Zr^4+^‐O cluster and contribute to the ESR signals, ultrafast transient absorption (TA) spectroscopy was used to investigate the kinetics for further electron transfer (i.e.*, V*_transfer‐2_).

A femtosecond UV pump/white light continuum (WLC) probe beam was used. The pump laser was chosen at 380 nm, which can effectively excite electrons from the CB to VB for UiO‐66‐NH_2_ (see UV–vis spectra in Figure S6, Supporting Information), and subsequent WLC probing was maintained in the range of 440–730 nm. Under different probe delays (Figure 2c), no essential variations in the spectral profiles were observed, and all the spectra exhibited a negative signal at 440–500 nm corresponding to the stimulated emission process, which is consistent with the photoluminescence (PL) emission spectra shown in Figure 2d.^[^ ^29^, ^30^ ^]^ However, the samples showed significant differences in the TA kinetics on comparing their excited state dynamics at 650 nm. All the kinetics traces can be fitted using a three‐exponential decay function (Figure 2e and **Table** **2**). Although an accurate assignment of these multiple time constants is challenging because they can arise from charge transfer or different trapping processes (e.g., shallow or deep trap states),^[^ ^30^ ^]^ since the co‐catalyst within the MOFs can be regarded as a deeper trap state due to its superior electron sink abilities, which can result in a very long relaxation time, it is reasonable to assign *τ*_3_, rather than *τ*_1_ and *τ*_2_, to the electron transfer process from the Zr‐O cluster to the co‐catalyst (i.e.*, V*_transfer‐2_). Subsequently, two conclusions can be drawn: i) The co‐catalyst can strengthen the electron transfer process between the Zr‐O cluster to the co‐catalyst, as indicated by the percentage change observed for *τ*_3_ [(UiO‐66‐NH_2_ (11.4%) < Pt/UiO‐66‐NH_2_ (14.2%) and PtPd polyhedrons/UiO‐66‐NH_2_ (15.8%)] and ii) the introduction of Pd in Pt can not only decrease the cost of the co‐catalyst, but can also accelerate *V*_transfer‐2_, as verified by the decreasing order observed for *τ*_3_ [UiO‐66‐NH_2_ (3633.7 ps) > Pt/UiO‐66‐NH_2_(2483.7 ps) > PtPd polyhedrons/UiO‐66‐NH_2_ (1772.9 ps)]. Interestingly, the results can also explain the ESR results because the enhanced *V*_transfer‐2_ can accelerate the change from Zr^3+^ to Zr^4+^; Pt/UiO‐66‐NH_2_ with a higher *V*_transfer‐1_ and lower *V*_transfer‐2_ has a significantly higher ESR intensity than PtPd polyhedrons/UiO‐66‐NH_2_.

**Table 2:** Summary of the TA parameters of the as‐prepared samples

	*τ*_1_/ps [pct.]	*τ*_2_/ps [pct.]	*τ*_3_/ps [pct.]	*τ*_average_/ps
UiO‐66‐NH_2_	83.4 (54.62%)	342.1 (33.98%)	3633.7 (11.4%)	576.0
Pt/UiO‐66‐NH_2_	3.1 (36.48%)	180.4 (49.32%)	2483.7 (14.2%)	442.8
PtPd polyhedrons/UiO‐66‐NH_2_	25.3 (46.16%)	243.5 (38.03%)	1772.9 (15.81%)	384.6
PtPd polyhedrons@UiO‐66‐NH_2_	2.1 (15.39%)	127.4 (65.26%)	1378.4 (19.35%)	350.2
PtPd octahedrons@UiO‐66‐NH_2_	5.8 (15.72%)	140.8 (64.27%)	1329.3 (20.01%)	357.4
PtPd cubes@UiO‐66‐NH_2_	4.9 (16.08%)	154.8 (64.15%)	1297.5 (19.77%)	356.6

More interestingly, in a 4 h H_2_ production test, PtPd polyhedrons/UiO‐66‐NH_2_ showed an ≈1.6‐ and ≈42.5‐fold enhancement in the H_2_ production rate (2.55 µmol h^−1^; Figure 2f and Table 1) when compared to Pt/UiO‐66‐NH_2_ (1.59 µmol h^−1^) and UiO‐66‐NH_2_ (0.06 µmol h^−1^) respectively, which suggests the introduction of Pd does not compromise the H_2_ generation activity of Pt, despite the fact that Pd is usually regarded as an unfavorable co‐catalyst due to its strong Pd—H bond.^[^ ^31^, ^32^ ^]^ The enhanced photoactivity also reflects the evaluation of the real H_2_ generation active sites (Table 1). Since the H_2_ molecules should be mainly generated on the surface of every single co‐catalyst particle, the TOF_specific_ value, defined as the number of H_2_ molecules generated on unit surface area of a single catalyst particle per minute, is used to show the H_2_ evolution ability of the co‐catalyst. The PtPd polyhedrons/UiO‐66‐NH_2_ catalyst generated 10.7 H_2_ molecules on the PtPd alloy per minute per square nanometer (i.e., 10.7 min^−1^ nm^−2^), which was 1.7 times larger than that of Pt/UiO‐66‐NH_2_ (6.4 min^−1^ nm^−2^).

The improved catalytic performance of PtPd polyhedrons/UiO‐66‐NH_2_ can be attributed to the significantly increased *V*_transfer‐2_ resulting from the additional electron transfer from Pd to Pt to equilibrate the electron Fermi distribution^[^ ^33^ ^]^ because of the larger work function of Pt compared to Pd.^[^ ^33^, ^34^, ^35^, ^36^ ^]^ The electron transfer from Pd to Pt was verified using X‐ray photoelectron spectroscopy (XPS) (Figure S8, Supporting Information). As can be seen in the high‐resolution spectra of the PtPd alloys, the binding energies of Pd 3d and Pt 4f shift to higher and lower values, respectively, when compared to those of the pure Pt and Pd particles, which demonstrate that some electrons are transferred from Pd to Pt atoms in the alloy structure,^[^ ^37^ ^]^ thus strongly supporting the MOF → Pd → Pt transfer pathway and efficient suppression of electron‐hole recombination. Our hypotheses can be proved by analyzing the average relaxation lifetime of TA (*τ*_a_) (Figure 2e and Table 2), PL emission spectra (Figure 2d), and photocurrent tests (Figure 2g). When compared with the parent UiO‐66‐NH_2_ catalyst, both Pt/UiO‐66‐NH_2_ and PtPd polyhedrons/UiO‐66‐NH_2_ show decreased relaxation times and PL intensities as well as enhanced photocurrent responses, which suggest the improved separation of the electron‐hole pairs on introduction of the co‐catalyst, irrespective of whether it is Pt or PtPd. Further, PtPd polyhedrons/UiO‐66‐NH_2_ shows a relatively decreased relaxation time, lower PL intensity, and higher photocurrent than Pt/UiO‐66‐NH_2_, which suggests the recombination of the electron‐hole pairs can be further inhibited when Pd is incorporated into Pt.

**Figure 3:**
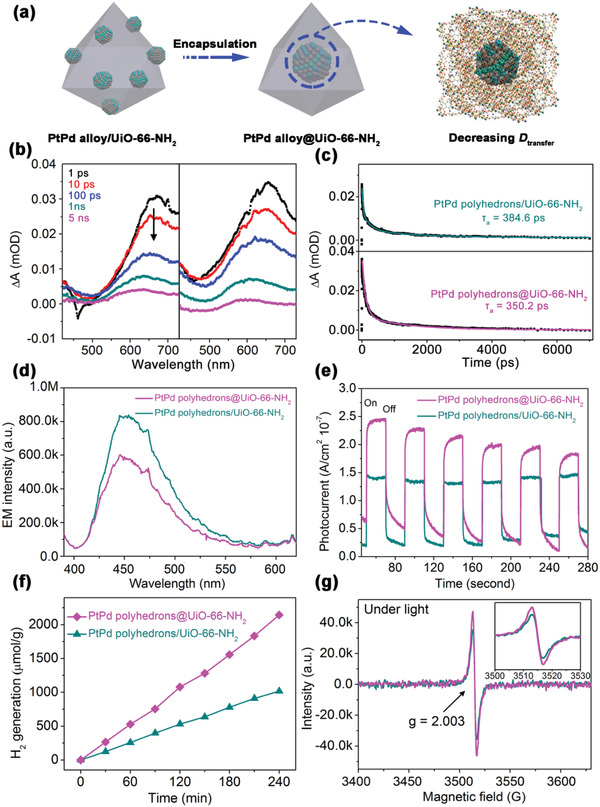
a) Schematic illustration of step 2, b) TA spectra pump at 380 nm at different delays in the range of 440−730 nm, c) TA kinetics observed at a wavelength of 650 nm and corresponding multi‐exponential fitting, d) steady‐state PL emission spectra excited at 380 nm, e) photocurrent measurements, f) H_2_ production performance, and g) ESR analysis under visible light irradiation for 5 min for PtPd polyhedrons/UiO‐66‐NH_2_ and PtPd polyhedrons@UiO‐66‐NH_2_.

According to Jiang's and our recent works,^[^ ^16^, ^38^ ^]^ the spatial positions of the co‐catalyst are manipulated to suppress any possible electron‐hole pair recombination during the transfer process in the second step (**Figure** **3**a). When charges are generated, they tend to transfer to the surface of the crystals to react with the reactants, but a long transfer distance (*D*_transfer_) will cause further electron‐hole recombination (i.e., unnecessary, but inevitable electron loss) to decrease the reaction efficiency. Fortunately, MOFs offer an opportunity to design a relatively shorter transfer pathway because their ordered porous structures permit the encapsulation of the co‐catalyst. Thus, a “bottle‐around‐ship” strategy was used to encapsulate the PtPd polyhedrons into the UiO‐66‐NH_2_ crystals (PtPd polyhedrons@UiO‐66‐NH_2_). Figure 1d shows that the encapsulated particles with an average size of 5.4 nm were well dispersed inside the MOFs crystals. The concentration of Pt and Pd in the PtPd polyhedron/UiO‐66‐NH_2_ and PtPd polyhedron@UiO‐66‐NH_2_ samples was determined using ICP‐MS and adjusted to be the same prior to comparing their H_2_ evolution performance.

Figure 3b,c can be used to compare the TA kinetics of the PtPd polyhedrons@UiO‐66‐NH_2_ and PtPd polyhedrons/UiO‐66‐NH_2_ samples. The position of the alloy had no significant effect on the spectral profile, but it significantly enhanced the *V*_transfer‐2_ (*τ*_3_ values: 1378.4 ps vs 1722.9 ps) due to the decreased *D*_transfer_. Thus, a significant inhibition of electron‐hole pair recombination was expected, which was proven by comparing the TA average relaxation lifetimes, PL emission intensities, and photocurrent intensities shown in Figure 3d,e. PtPd polyhedrons@UiO‐66‐NH_2_ possesses a smaller *τ*_a_ value of 350.2 ps (Table 2), weaker PL intensity, but higher photocurrent response than PtPd polyhedrons/UiO‐66‐NH_2_. The enhanced separation in the encapsulation structure is also reflected by the H_2_ production. Figure 3f shows PtPd polyhedron@UiO‐66‐NH_2_ exhibits a higher H_2_ generation rate and calculated TOF_specific_ value compared to polyhedron/UiO‐66‐NH_2_ (5.36 and 22.1 vs 2.55 and 10.7 min^−1^ nm^−2^, respectively). It should be noted that although an enhanced *V*_transfer‐2_ normally leads to a decreased ESR intensity because Zr^3+^ changes back to Zr^4+^, the slightly increased ESR signal observed in Figure 3g can be attributed to the enhanced *V*_transfer‐1_ of PtPd polyhedrons@UiO‐66‐NH_2_. This was confirmed by the significantly enhanced visible light absorption observed in the UV–vis spectrum of PtPd polyhedrons@UiO‐66‐NH_2,_which showed the decreased *E*_g_ value, thus indicating the enhanced photoexcited process.

These results inspired us to consider a third step to increase *V*_consume_ by manipulating the PtPd alloy shape because it is known that the facets or shapes of the co‐catalysts play an essential role in reactant adsorption and activation performance, which can significantly influence the photoreduction efficiency.^[^ ^39^, ^40^ ^]^ This necessity also lies in the fact that an appropriate facet selection can maximize the noble metal utilization efficiency. Figure 1b,c shows the different morphologies of the PtPd nanocrystals prepared with different facets. The synthesis strategy was similar to that used to prepare the PtPd polyhedrons using a fixed 1:1 molar ratio of the Pd and Pt precursors and a reduction temperature of 160 °C except for the selection of different capping agents. For the synthesis of PtPd octahedrons with an edge length (*d*_2_) of 6.59 nm (Figure 1b), an appropriate amount of HCHO was used to expose the (111) facets.^[^ ^41^ ^]^ PtPd cubes with an average edge length (*d*_3_) of 7.8 nm and exposed (100) facets (Figure 1c) required both Br^−^ and I^−^ as capping agents, whilst keeping the other conditions fixed.^[^ ^42^ ^]^ All of the as‐prepared PtPd alloys exhibited Pt/Pd molar ratios ≈ 1, which was consistent with the precursor molar ratio used. The amount of encapsulated PtPd alloy was determined using ICP‐MS to ensure the total amount of metal was the same in each sample. It is interesting to note that during the encapsulation process, an “interfacial energy barrier” or “lattice mismatch effect” between the MOFs and PtPd alloys was not observed, especially when there are several reports of the occurrence of severe self‐nucleation when guests with sharp corners and edges are used.^[^ ^43^, ^44^, ^45^ ^]^ Instead, the immobilization process was easily accomplished and the resulting samples are shown in Figure 2e,f. The encapsulated structures all exhibit good crystallization and no changes occur in terms of their texture when compared with pristine UiO‐66‐NH_2_ (see XRD patterns in Figure S9, Supporting Information). The TEM images show that within the UiO‐66‐NH_2_ octahedrons, the PtPd octahedrons and cubes are well dispersed as polyhedrons without particles outside the crystals. Moreover, the UV‐diffuse spectra (Figure S6, Supporting Information) show that the encapsulated structures exhibit enhanced light adsorption in the range of 450–800 nm when compared to the PdPt alloys on the outside, which was also consistent with the grey colored sample instead of a mixture of black and yellow (Figure S10, Supporting Information). Therefore, we preliminarily deduced that i) the overall particle sizes are below 10 nm during the encapsulation process and ii) the introduction of Pd into Pt with the PVP surfactant^[^ ^46^ ^]^ somehow decreases the lattice mismatch between the metal and UiO‐66‐NH_2_.

**Figure 4:**
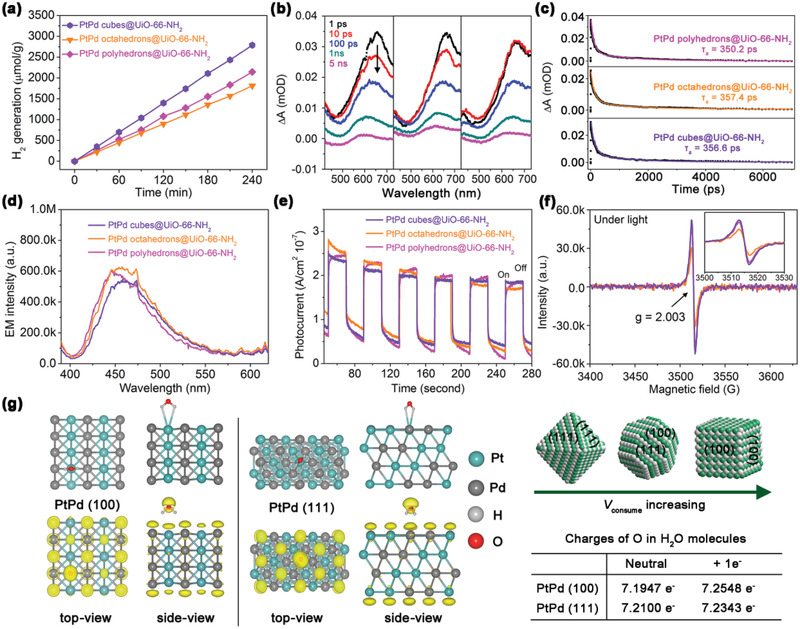
a) H_2_ production performance, b) TA spectra pump at 380 nm at different delays in the range of 440−730 nm, c) TA kinetics observed at a wavelength of 650 nm and corresponding multi‐exponential fitting, d) steady‐state PL emission spectra excited at 380 nm, e) photocurrent measurements, and f) ESR analysis under visible light irradiation for 5 min for PtPd polyhedrons@UiO‐66‐NH_2_, PtPd octahedrons@UiO‐66‐NH_2_, and PtPd cubes@UiO‐66‐NH_2_. The left of (g) is the model used in our first‐principles simulations; the right is a schematic representation of Step 3 used to control *V*_consume_ and a summary of the charges on O in H_2_O using PtPd (100) and PtPd (111), respectively.

In addition, 4‐h H_2_ production tests (**Figure** **4**a) showed that the H_2_ generation rate using PtPd octahedrons@UiO‐66‐NH_2_ with (111) facets was lower than that of PtPd polyhedrons@UiO‐66‐NH_2_ with mixed (100) and (111) facets (4.53 vs 5.36 µmol h^−1^). When (100) facets are present, the H_2_ generation rate and calculated TOF_specific_ value increase to 6.97 µmol h^−1^ and 42.6 min^−1^nm^−2^, which are 1.3‐ and 1.54‐times as well as 1.9‐ and 2.3‐times higher than those observed for the polyhedrons and octahedrons, respectively. However, the enhanced activity is unlikely due to an enhanced charge separation ability because all of the encapsulated structures have similar *τ*_3_ values and percentages, as well as *τ*_a_ values (Figure 4b,c, *τ*_3_ values: 1378.4, 1329.3, 1297.5 ps; percentages: 19.3%, 20.0%, 19.77%; *τ*_a_ values: 350.2, 357.4, 356.6 ps for polyhedrons, octahedrons, and cubes, respectively). This is also supported by their comparable PL (Figure 4d) and photocurrent intensities (Figure 4e), which indicates that the facets of the PdPt alloy have a very limited contribution to inhibiting the electron and hole recombination. Therefore, we deduce that the dramatic differences in the H_2_ production efficiency may result from the differences in the *V*_transfer‐1_ and *V*_consume_ values. For *V*_transfer‐1_, evidence is available from the UV–vis and ESR spectra (Figure 4f). The E_g_ value, which shows the following order: cubes < polyhedrons < octahedrons, suggests that *V*_transfer‐1_ increases when changing the facets from (111) to (100). In addition, since under the same *V*_transfer‐2_, the ESR intensity, which shows the following order: cubes > polyhedrons > octahedrons, was highly dependent on *V*_transfer‐1_ and is thus consistent with the UV spectra.

We then applied first‐principles simulations to investigate the electron transfer behavior toward H_2_O (i.e.*, V*_consume_, see Experimental Section for Model construction process and calculation details). As summarized in Figure 4g, under neutral conditions with one additional electron introduced (simulating the injection of one photoelectron), the PtPd alloy with (111) facets exhibits a higher absolute adsorption energy (*E*_a_) of −0.193 and −0.308 eV toward H_2_O when compared to PtPd (100) (−0.192 and −0.273 eV), which is consistent with that previously reported.^[^ ^31^ ^]^ However, with one additional electron, ≈0.024 extra e^−^ transfers to the O atom in H_2_O adsorbed on PtPd (111) when compared to that under neutral conditions, which is ≈2.5‐times lower than the situation when H_2_O was adsorbed on the PtPd (100) facet (extra ≈0.06 e^−^). These results suggest that the PtPd (111) facet has a relatively low *V*_consume_, or in other words, weaker H_2_O molecule activation ability than PtPd (100). Therefore, under a similar electron‐hole pair separation situation, the low *V*_consume_ decreases the H_2_ generation rate. On the other hand, a relatively higher *V*_consume_ can accelerate H_2_ generation, which is consistent with the present H_2_ production activity. Taken together, these results highlight the importance of constructing high *V*_consume_ and high *V*_transfer_ Schottky junctions to enhance the photoactivity of MOFs.

Life span tests on PtPd cube@UiO‐66‐NH_2_ demonstrate that no significant changes occur during the production of H_2_ after four runs (Figure S11, Supporting Information). The corresponding XRD pattern shown in Figure S12, Supporting Information indicates that the structural integrity and crystallinity are retained when compared to the fresh sample. On the contrary, the H_2_ evolution performance over Pt/UiO‐66‐NH_2_ shows a dramatic decrease after four cycles (from 1.59 to 0.87 µmol h^−1^, a ≈45% decrease). TEM characterization of the morphology shows no significant aggregation of the PtPd cubes inside UiO‐66‐NH_2_ after recycling (Figure S13, Supporting Information). The cubes are well dispersed and have no morphological changes. Moreover, the same metal concentration in PtPd cube@UiO‐66‐NH_2_ (0.25 and 0.13 wt% for Pt and Pd, respectively) confirmed using ICP‐MS before and after the reactions was in contrast to the significant loss of Pt observed in Pt/UiO‐66‐NH_2_ (0.50 vs. 0.36 wt%), which illustrates the good co‐catalyst fixability of the designed junction.

## 3 Conclusion

We constructed an efficient Schottky junction using three steps to optimize the *V*_transfer_, *D*_transfer_, and *V*_consume_, respectively: (i) Partial substitution of Pt with Pd to form PtPd alloy, (ii) encapsulation of PtPd into the MOFs crystals, and (iii) controlling the shape of the inner alloy to expose the (100) facets. *V*_transfer_, *D*_transfer_, and *V*_consume_ had significant synergistic effects on the newly designed structure, PtPd cubes@UiO‐66‐NH_2_. When compared to pristine UiO‐66‐NH_2_ and the traditional Schottky junction (Pt/UiO‐66‐NH_2_), the designed structure with high *V*_consume_ and *V*_transfer‐2_ showed a >116‐ and 4.4‐fold enhancement in H_2_ generation, respectively.

## 4 Experimental Section

##### Materials

Zirconium(IV)chloride (ZrCl_4_), palladium(II)chloride (PdCl_2_), hydrogen hexachloroplatinate(IV)hexahydrate (H_2_PtCl_6_•6H_2_O), potassium hexachloroplatinate(IV) (K_2_PtCl_6_), TEOA, and hydrochloric acid (HCl, 37%) were supplied by Aladdin Industrial Corporation (Shanghai). 2‐Aminoterephthalic acid (BDC‐NH_2_) was supplied by Alfa Aesar. N,N‐dimethylformamide (DMF) and acetic acid were purchased from Tianjin Fuyu Fine Chemical Co., Ltd. Methanol (99.8%) and acetone were purchased from Beijing Chemical Works. Polyvinylpyrrolidone (MW = 55 000), potassium bromide (KBr), sodium chloride (NaCl), potassium iodide (KI), and formaldehyde solution (HCHO, 40%) were purchased from Sinopharm Chemical Reagent Co., Ltd. All chemical reagents were used as received without further purification.

##### Preparation of 5.4 nm Pt Particles

5.4 nm Pt particles were prepared using the following method:^[^ ^47^ ^]^ 40 mg of PVP was dissolved in 2.0 mL of EG and then placed into a 20 mL glass vial. The vial was then placed into an oil bath preheated at 160 °C under magnetic stirring. Then, 1.0 mL of K_2_PtCl_6_ solution in EG (16 mg mL^−1^) was quickly injected into the vial and reacted for 1 h. After cooling to the room temperature, the as‐synthesized PVP‐protected Pt particles were precipitated using acetone, washed three times with acetone/hexane to remove the excess free PVP, and re‐dispersed in DMF to give a colloidal solution of the Pt NPs with a concentration of ≈1 mg mL^−1^.

##### Preparation of 5.4 nm PtPd Polyhedrons

0.0146 g of K_2_PtCl_6_, 0.0053 g of PdCl_2_, 0.1 g of PVP (MW = 55 000), and 0.357 g of KBr were dissolved in 10 mL of water heated at 50 °C with stirring. Then, 20 µL of formaldehyde solution (40%) was added and stirred for 10 min. The solution was transferred into a 25 mL Teflon‐lined stainless‐steel autoclave and heated at 160 °C for 3 h. After cooling to room temperature, the products were washed three times using acetone and ethanol via centrifugation, and the PtPd polyhedrons redispersed in 10 mL of DMF.

##### Preparation of PtPd Octahedrons

0.0146 g of K_2_PtCl_6_, 0.0053 g of PdCl_2_, 0.1 g of PVP (MW = 55 000), and 0.357 g of KBr were dissolved in 10 mL of water heated at 50 °C with stirring. Then, 50 µL of formaldehyde solution (40%) was added and stirred for 10 min. The solution was transferred into a 25 mL Teflon‐lined stainless‐steel autoclave and heated at 160 °C for 3 h. After cooling to room temperature, the products were washed three times using acetone and ethanol via centrifugation and the PtPd octahedrons redispersed in 10 mL of DMF.

##### Preparation of PtPd Cubes

According to a literature procedure with some modification,^[^ ^42^ ^]^ 0.0146 g of K_2_PtCl_6_, 0.0053 g of PdCl_2_, 0.357 g of KBr, and 100 mg of PVP (MW = 55 000) were dissolved in 10 mL of water heated at 50 °C with stirring. Then, 0.5 mL of KI (0.001 g) solution was added and stirred for 10 min. The solution was transferred to a 25 mL Teflon‐lined stainless‐steel autoclave and heated at 160 °C for 3 h. After cooling to room temperature, the products were washed three times using acetone and ethanol via centrifugation and the PtPd cubes redispersed in 10 mL of DMF.

##### Preparation of UiO‐66‐NH_2_ Octahedrons

According to a literature procedure with some modification,^[^ ^10^ ^]^ ZrCl_4_ (0.233 g) and 2‐aminoterephthalic acid (0.181 g) were dissolved in 102.1 mL of DMF. Then, acetic acid (22.9 mL) was added to the solution, followed by sonication for 10 min. The solution was then transferred into a 200 mL Teflon‐lined stainless‐steel autoclave and heated to 120 °C for 16 h. After cooling to room temperature, the products were collected by centrifugation, washed three times using DMF to remove the excess reactants, washed three times using methanol to remove the DMF, and heated at 150 °C under vacuum for 12 h.

##### Preparation of 5.4 nm PtPd Polyhedrons, Octahedrons, and Cubes@UiO‐66‐NH_2_

The preparation of 5.4 nm PtPd polyhedrons, octahedrons, and cubes@UiO‐66‐NH_2_ was similar to the preparation of UiO‐66‐NH_2_ octahedrons except for the addition of a certain amount of the PtPd polyhedrons/octahedrons/cubes dispersion into the precursor solution and well‐dispersed via sonication before being heated at 120 °C.

##### Photocurrent Tests

Photoelectrochemical measurements were performed on a CHI 660E electrochemical workstation using a standard three‐electrode system with the photocatalyst‐coated FTO as the working electrode, Pt plate as the counter electrode, and Ag/AgCl as the reference electrode. A 300 W Xenon lamp was used as the light source. A 0.5 m Na_2_SO_4_ solution was used as the electrolyte. The samples (2 mg) were added to a mixed solution comprised of Nafion (10 µL) and ethanol (2 mL), and the working electrodes were prepared by dropping the suspension (100 µL) onto the surface of an FTO plate. The working electrodes were dried at 80 °C.

##### Photocatalytic H_2_ Production

Photocatalytic H_2_ production reactions were carried out in a 200 mL closed quartz flask reactor. 10 mg of the photocatalyst was dispersed in 101 mL of a mixed solution comprised of CH_3_CN, H_2_O, and TEOA with a volume ratio of 90:1:10. Before irradiation, the reactor was degassed for 1 h to drive away any residual air. Photocatalytic hydrogen production was then triggered by irradiating the suspension using a 300 W of xenon lamp (PLS‐SXE300C). The gas products were analyzed periodically using a gas chromatograph (GC9790II) equipped with a TCD detector.

##### Characterization

TEM images were obtained on a JEOL‐1011 transmission electron microscope. Powder XRD (Rigaku Ultima IV) was used to characterize the crystal structures of the materials. The chemical components and state were examined using XPS (SHIMADZU AXIS Supra). UV–vis diffuse reflectance spectroscopy (Cary 5000) was used to measure the light absorption of the samples. PL spectroscopy (FLS980) was used to investigate the recombination of the photogenerated electron‐hole pairs and the PL emission spectra was obtained. In situ ESR measurements were recorded using electron paramagnetic resonance spectroscopy on a BrukerA300‐10/12 spectrometer. The typical procedure was performed as follows: 10 mg of sample was dispersed in a mixture comprised of CH_3_CN, water, and TEOA (same volume ratio as used in the H_2_ evolution test) and sonicated for 10 min. The signals were collected in the dark, the 300 W xenon lamp (PLS‐SXE300C) was turned on, and the signals collected after the sample solution was irradiated for 5 min (start timing when the light was switched on).

##### TA Analysis

The pump beam was generated from a regenerative amplified Ti:sapphire laser system from Coherent (800 nm, 100 fs, 6 mJ per pulse, and 1 kHz repetition rate). The 800 nm output pulse from the regenerative amplifier was split in two parts using a beam splitter. The reflected part was used to pump a TOPAS optical parametric amplifier, which generated a wavelength‐tunable laser pulse from 250 nm to 2.5 µm as the pump beam. The transmitted 800 nm beam was attenuated with a neutral density filter and was focused into a rotating CaF_2_ disk to generate a WLC from 350 to 800 nm and used as the probe beam. The probe beam was focused with an Al parabolic reflector onto the sample. After sampling, the probe beam was collimated and then focused into a fiber‐coupled spectrometer and detected at a frequency of 1 KHz. The intensity of the pump pulse used in the experiment was controlled using a variable neutral‐density filter wheel. The delay between the pump and probe pulses was controlled using a motorized delay stage. The pump pulses were chopped by a synchronized chopper at 500 Hz.^[^ ^48^ ^]^ All experiments were performed at room temperature. Typically, the catalysts were dispersed in MeCN:TEOA:water = 90:10:1 (volume ratio).

##### First‐Principles Calculations

The surfaces of PtPd (100) and PtPd (111) were modeled using slab models consisting of $2\sqrt{2}\times 2\sqrt{2}$ and $2\times 2\sqrt{3}$ cells in the x‐ and y‐direction and 4 layers of Pd and Pt atoms in the z‐direction with a vacuum of 15 Å in the z‐direction. First‐principles calculations were carried out using the VASP code^[^ ^49^, ^50^, ^51^, ^52^ ^]^ generalized gradient approximation for the exchange‐correlation functional and dispersion correction using Grimme's DFT‐D3 scheme^[^ ^53^, ^54^, ^55^ ^]^ utilized for the valence‐core interactions. During the structural optimization, the above two layers of atoms were allowed to relax until the forces on any atom became <0.01 eV Å^−1^. The convergence criterion of the self‐consistent calculations for ionic relaxations was 10^−5^ eV between two consecutive steps. The cut‐off energy for the plane wave basis was set at 500 eV. The first Brillouin zone was sampled using a *γ*‐centered method to generate k‐point meshes. A k‐point grid of 4 × 4 × 1 and 17 × 17 × 1 was used in the structural optimization and in the static calculations, respectively. The adsorption energies for H_2_O on the PtPd (100) and PtPd (111) surfaces were calculated using the following formula: *E*_a_ = E(PtPd‐H_2_O) − E(PtPd) − E(H_2_O), where E(PtPd‐H_2_O), E(PtPd), and E(H_2_O) are the DFT total energies of the H_2_O adsorbed PtPd surface, cleaned PtPd surface, and H_2_O molecule, respectively. The negative adsorption energy showed that H_2_O molecules can be stably adsorbed on the surface of PtPd.

##### Statistical Analysis

The experimental results for the TEM images are shown as raw data and no data pre‐processing was used. The measured particle sizes were presented as their average values.

## Conflict of Interest

The authors declare no conflict of interest.

## Supporting information

Supporting Information

## Data Availability

The data that supports the findings of this study are available in the supplementary material of this article
