# Supplementary material for: Efficient Schottky Junction Construction in Metal‐Organic Frameworks for Boosting H2 Production Activity
Source: Adv Sci (Weinh). 2021 May 7;8(13):2004456. doi: 10.1002/advs.202004456 (PMC8261486; doi:10.1002/advs.202004456)
Supplement: Supplementary file 1 — Supporting Information [file ADVS-8-2004456-s001.pdf]

© 2021 Wiley-VCH GmbH

ADVANCED SCIENCE
Open Access

Supporting Information

for *Adv. Sci.*, DOI: 10.1002/advs.202004456

Efficient Schottky Junction Construction in Metal-Organic Frameworks for Boosting H_2 Production Activity

Yang Wang, Wei Zhang, Dan Li, Jianping Guo, Yu Yu, Kejian Ding, Wubiao Duan, Xiyou Li, Heyuan Liu, Pengkun Su, Bo Liu,* and Jianfeng Li**

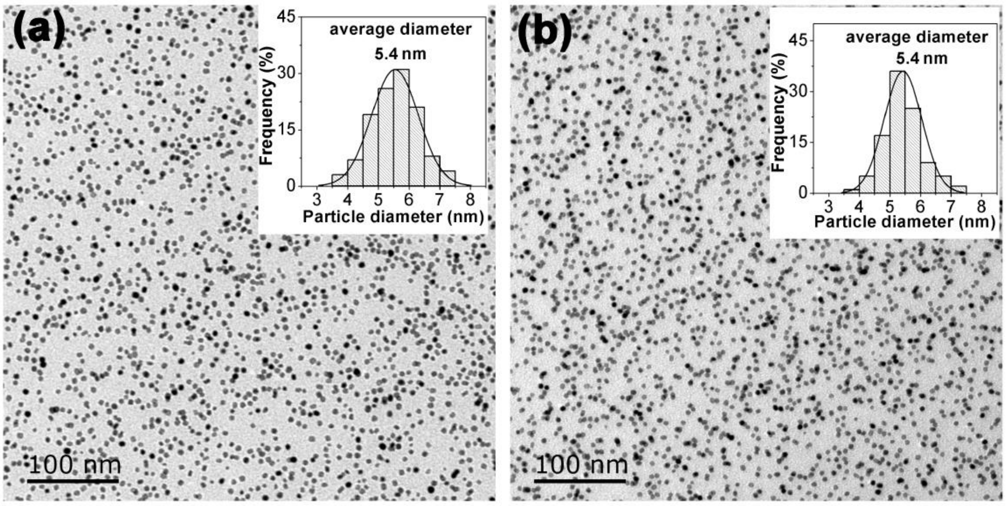

Figure S1 TEM images of PtPd octahedrons with an average diameter of 5.4 nm (a) and Pt particles with an average diameter of 5.4 nm (b). Inset (a) and (b) are the corresponding particle size distribution diagrams.

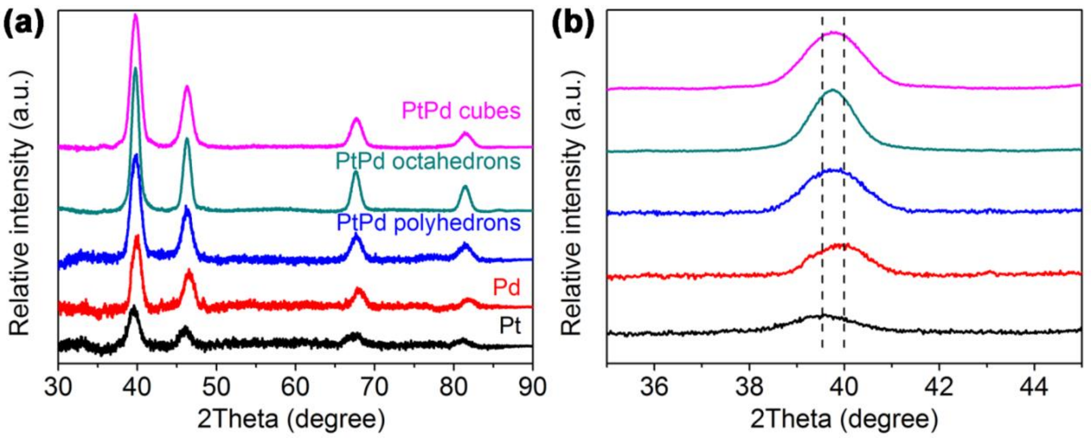

Figure S2 XRD patterns of Pt, Pd, PtPd polyhedrons, PtPd octahedrons, and PtPd cubes (a). (b) The (111) facet of the as-prepared particles in (a).

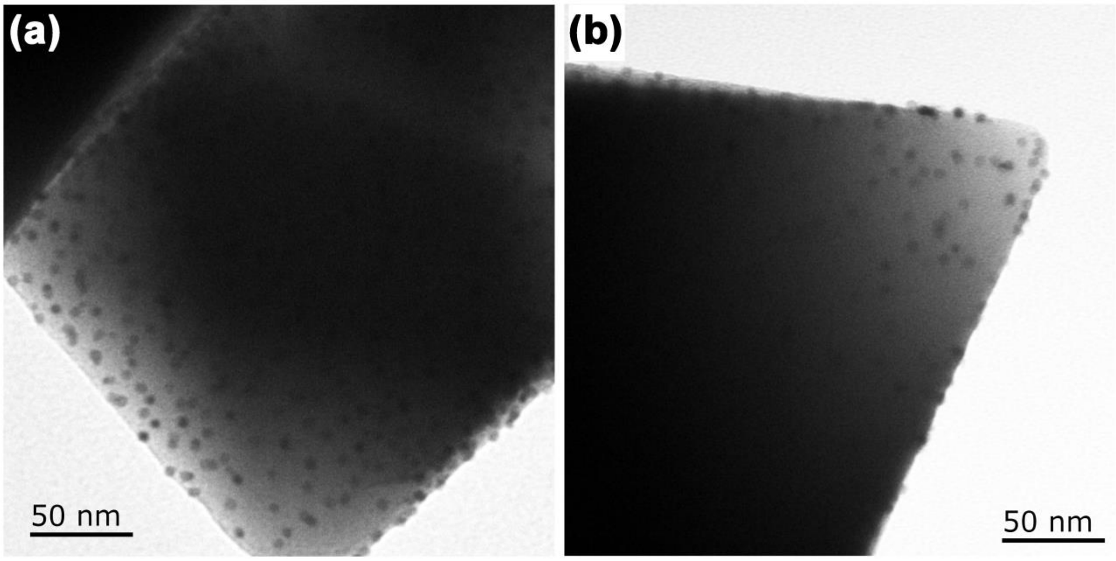

Figure S3 TEM images of 5.4 nm PtPd octahedrons/UiO-66-NH_2 and Pt/UiO-66-NH_2.

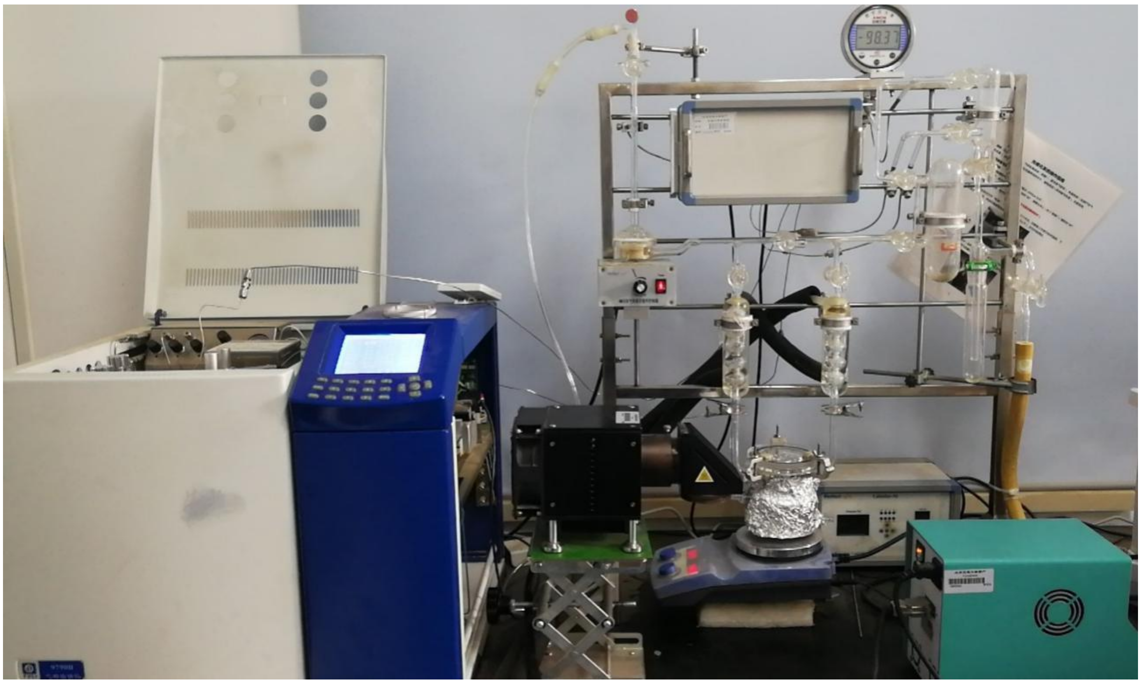

Figure S4 Photograph of the photocatalytic system used in our study.

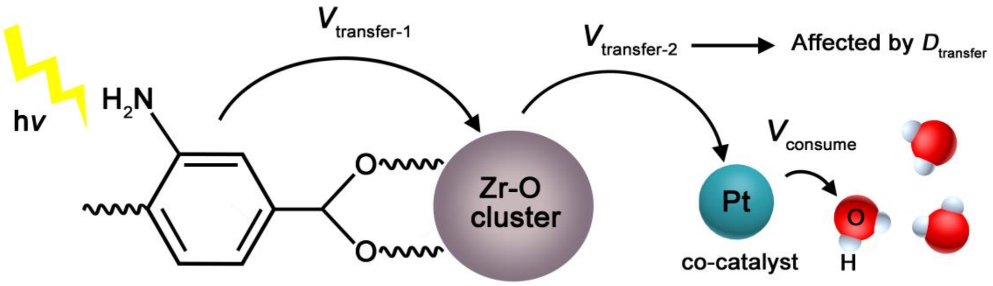

Figure S5 Proposed electron transfer process involving $V_{transfer}$, $D_{transfer}$, and $V_{consume}$.

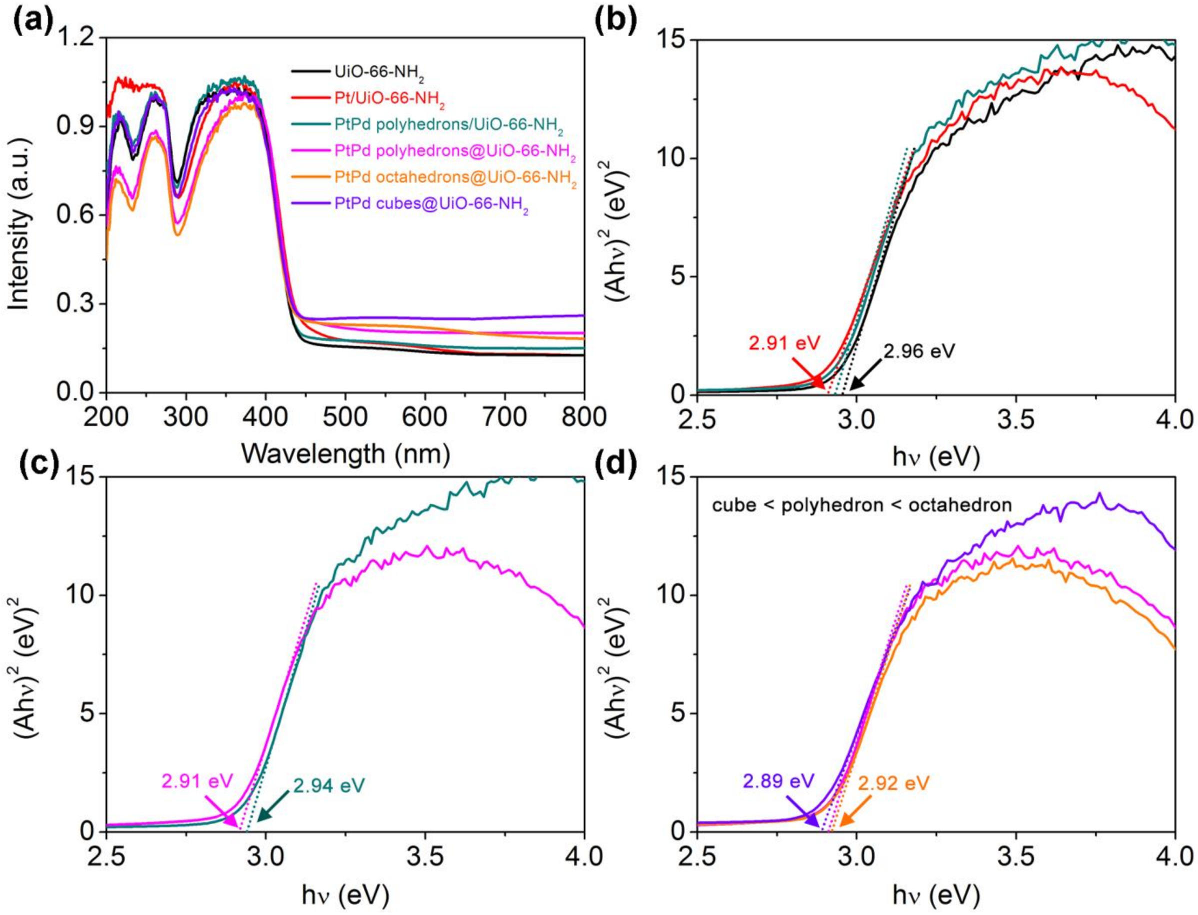

Figure S6 UV-Vis diffuse reflectance spectra and Tauc plots of UiO-66-NH_2, Pt/UiO-66-NH_2, PtPd polyhedrons/UiO-66-NH_2, PtPd polyhedrons@UiO-66-NH_2, PtPd octahedrons@UiO-66-NH_2, and PtPd cubes@UiO-66-NH_2.

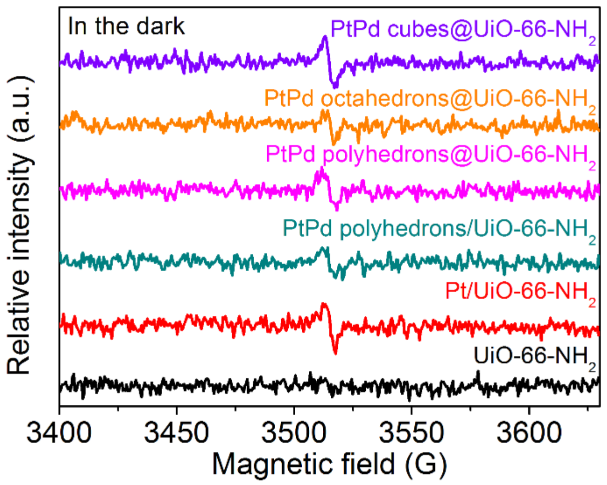

Figure S7 ESR signals collected in the dark for the as-prepared samples.

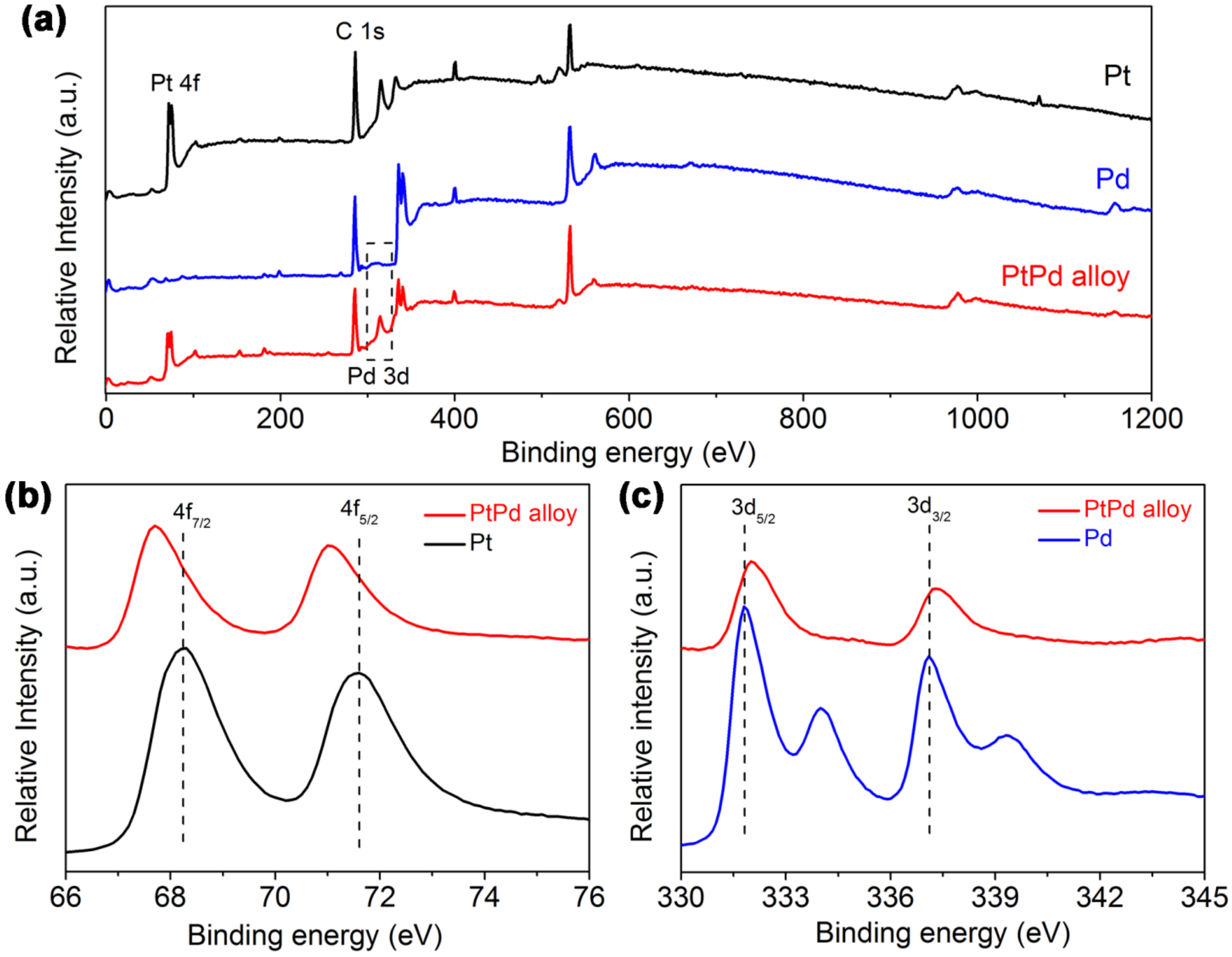

Figure S8 XPS survey spectra in the range of –1–1200 eV (a) and high-resolution spectra of the Pt 4f and Pd 3d for Pt, Pd, and PtPd alloy samples, respectively (b, c).

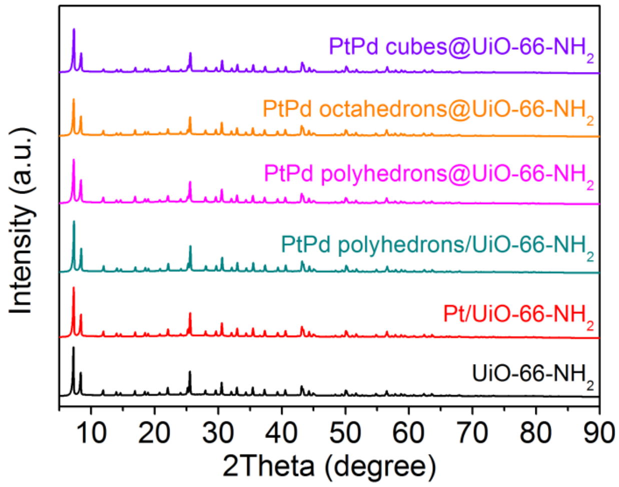

Figure S9 XRD patterns of UiO-66-NH_2, Pt/UiO-66-NH_2, PtPd polyhedrons/UiO-66-NH_2, PtPd polyhedrons@UiO-66-NH_2, PtPd octahedrons@UiO-66-NH_2, and PtPd cubes@UiO-66-NH_2.

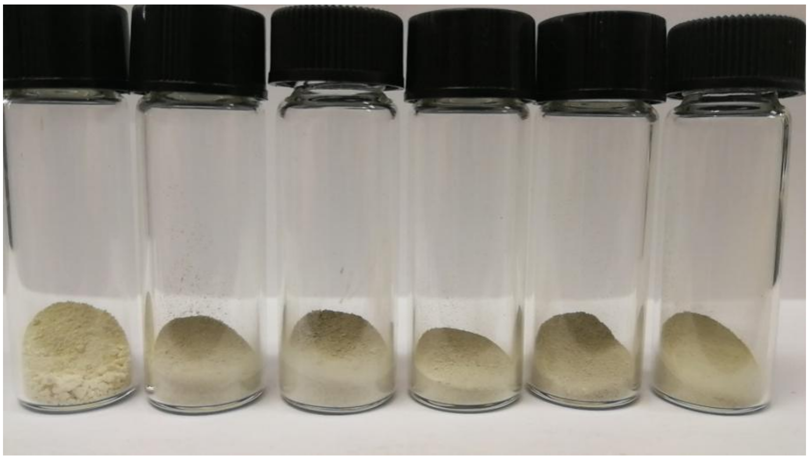

Figure S10 Photograph of the as-prepared samples. From left to right: UiO-66-NH_2, Pt/UiO-66-NH_2, PtPd polyhedrons/UiO-66-NH_2, PtPd polyhedrons@UiO-66-NH_2, PtPd octahedrons@UiO-66-NH_2, and PtPd cubes@UiO-66-NH_2.

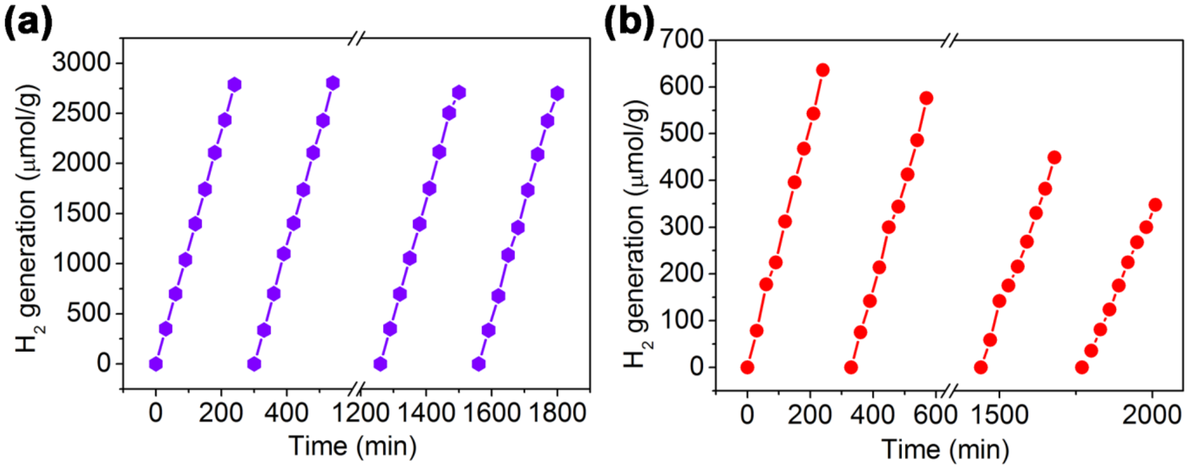

Figure S11 Life span tests for PtPd cube@UiO-66-NH_2 (a) and Pt/UiO-66-NH_2 (b).

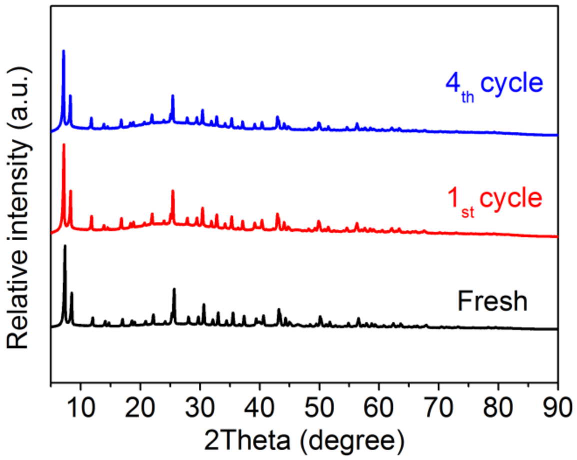

Figure S12 XRD patterns of PtPd cube@UiO-66-NH_2 before and after the tests.

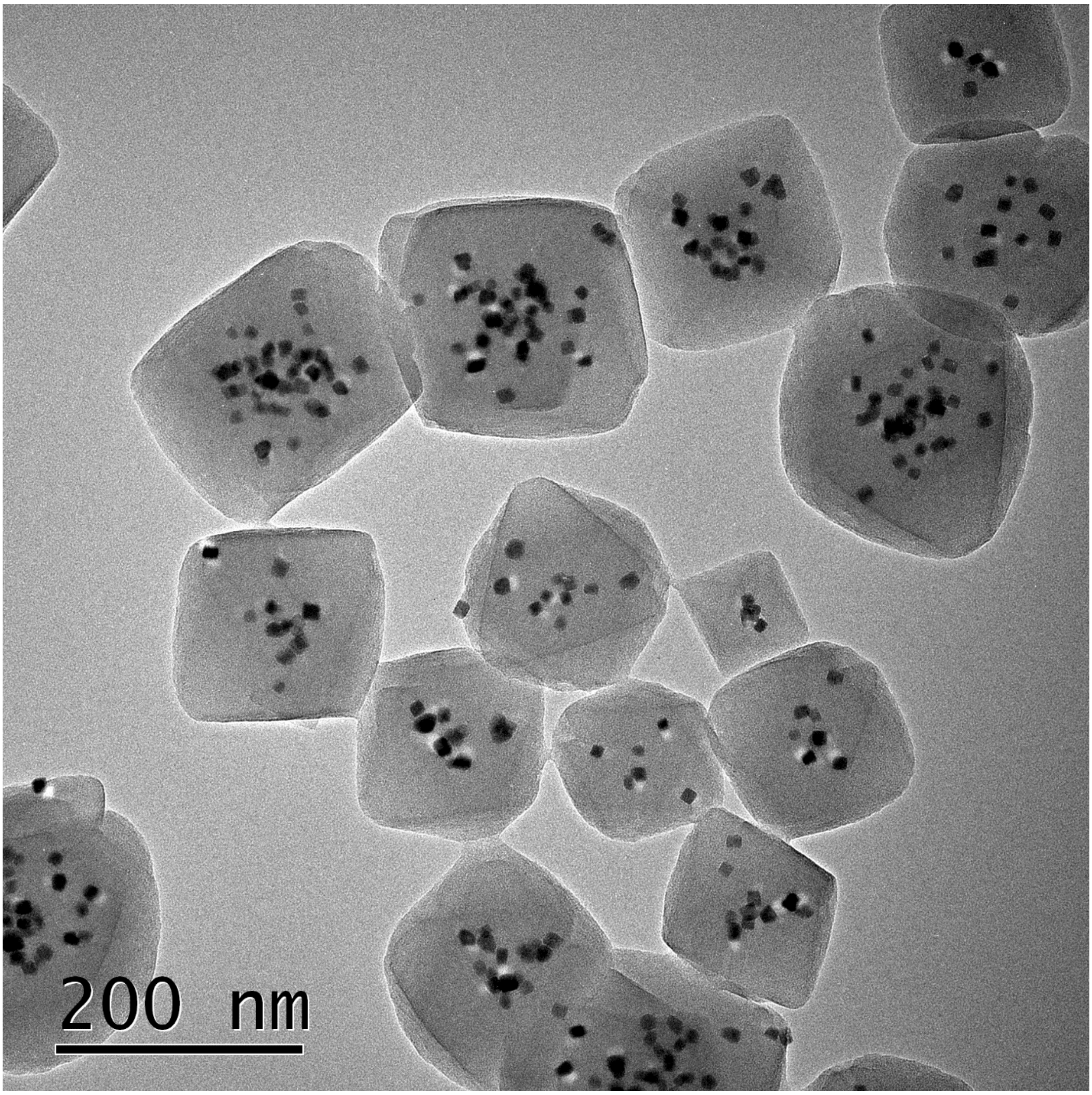

Figure S13 TEM images of PtPd cube@UiO-66-NH_2 after the tests.
